# Supplementary material for: A BET family protein degrader provokes senolysis by targeting NHEJ and autophagy in senescent cells
Source: Nat Commun. 2020 Apr 22;11:1935. doi: 10.1038/s41467-020-15719-6 (PMC7176673; doi:10.1038/s41467-020-15719-6)
Supplement: Supplementary file 1 — Supplementary Information [file 41467_2020_15719_MOESM1_ESM.pdf]

A BET family protein degrader provokes senolysis by targeting NHEJ and autophagy in senescent cells

Wakita et al.

Supplementary Fig.1

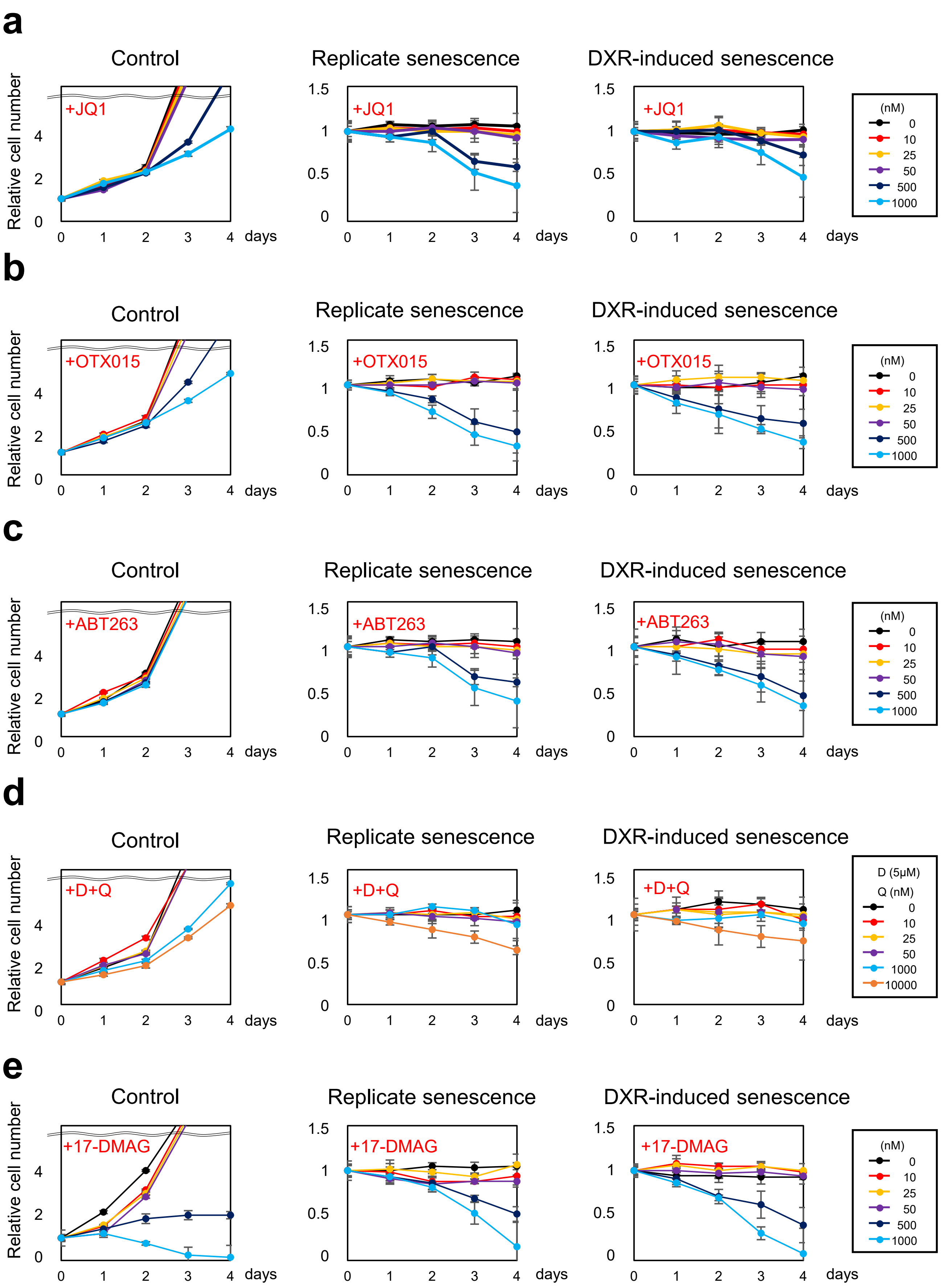

Supplementary Figure 1

Senolytic activity of various chemicals. Early passage pre-senescent (control) TIG-3 cells were rendered senescent by serial passage (replicative senescence) or treatment with 250 ng/ml DXR for 10 days (DXR-induced senescence). These senescent cells and control pre-senescent cells were incubated with each chemical (**a**, JQ1; **b**, OTX015; **c**, ABT263; **d**, the combination of dasatinib and quercetin; **e,** 17-DMAG) at the concentration shown right for 4 days. The relative cell number was counted throughout the experiments. For all graphs, error bars indicate mean ± s.d. and the representative date from three independent experiments was shown.

Supplementary Fig.2

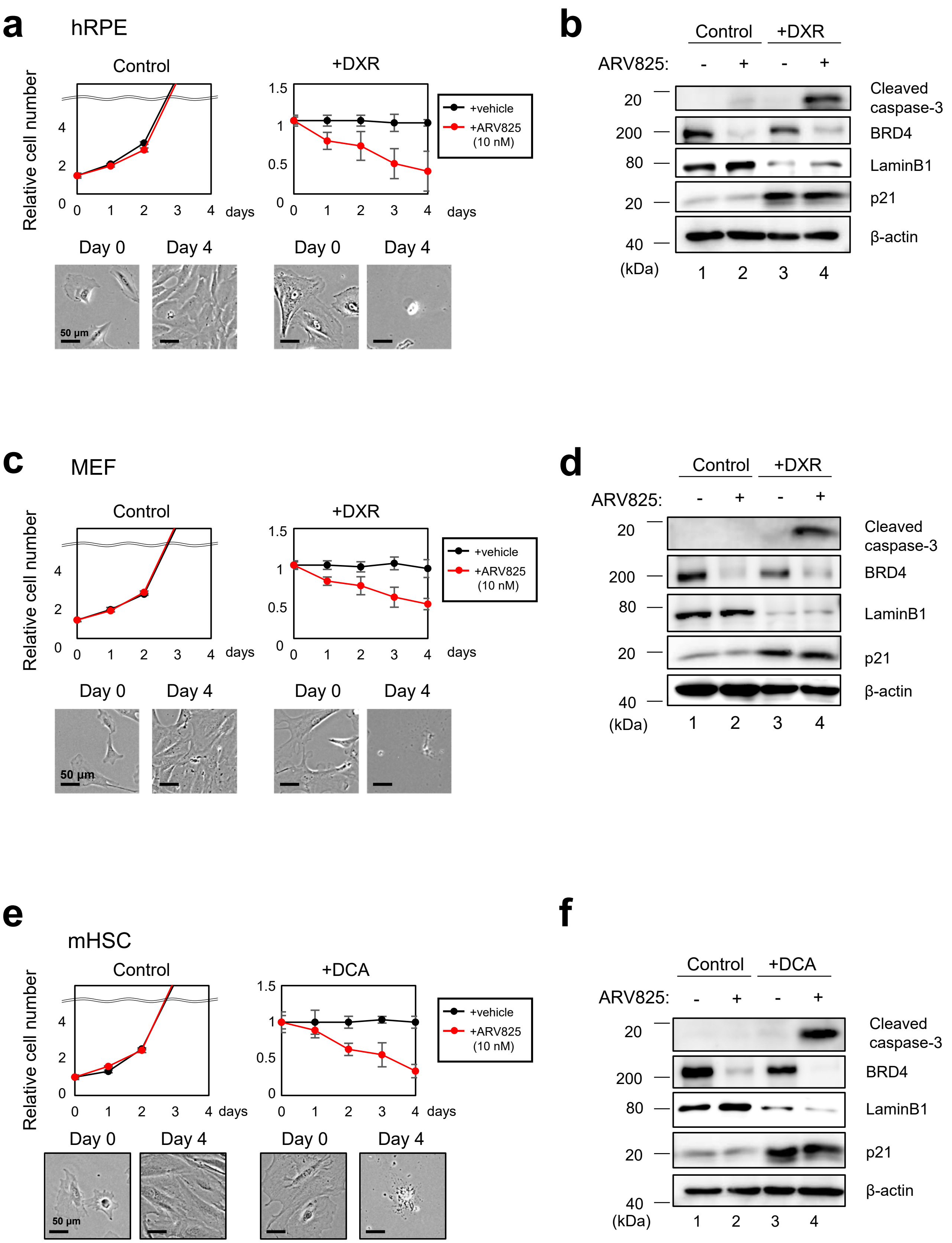

Supplementary Figure 2

ARV825 provokes senolysis across cell types and species. (**a and b**), Pre-senescent primary normal human retinal pigment epithelial (hRPE) cells were rendered senescent by treatment with 100 ng/ml DXR for 10 days. These senescent cells and control pre-senescent cells were treated with 10 nM ARV825 or vehicle for 4 days. Relative cell number was counted throughout the experiments and representative photographs of the cells in the indicated culture conditions are shown at the bottom of the panels (**a**). Cells treated with 10 nM ARV825 (+) or vehicle (-) for 4 days were subjected to western blotting using the antibodies shown on the right (**b**). (**c and d**), Pre-senescent primary mouse embryonic fibroblasts (MEFs) were rendered senescent by treatment with 100 ng/ml DXR for 10 days. These senescent cells and control pre-senescent cells were treated with 10 nM ARV825 or vehicle for 4 days. Relative cell number was counted throughout the experiments and representative photographs of the cells in the indicated culture conditions are shown at the bottom of the panels (**c**). Cells treated with 10 nM ARV825 (+) or vehicle (-) for 4 days were subjected to western blotting using the antibodies shown on the right (**d**). (**e and f**), Pre-senescent primary mouse hepatic stellate cells (HSCs) were rendered senescent by treatment with 275 μM DCA for 10 days. These senescent cells and control pre-senescent cells were treated with or without 10 nM ARV825 or vehicle for 4 days. Relative cell number was counted throughout the experiments and representative photographs of the cells in the indicated culture conditions are shown at the bottom of the panels (**e**). Cells treated with 10 nM ARV825 (+) or vehicle (-) for 4 days were subjected to western blotting using the antibodies shown on the right (**f**). For all graphs, error bars indicate mean ± s.d. and the representative date from three independent experiments was shown.

Supplementary Fig.3

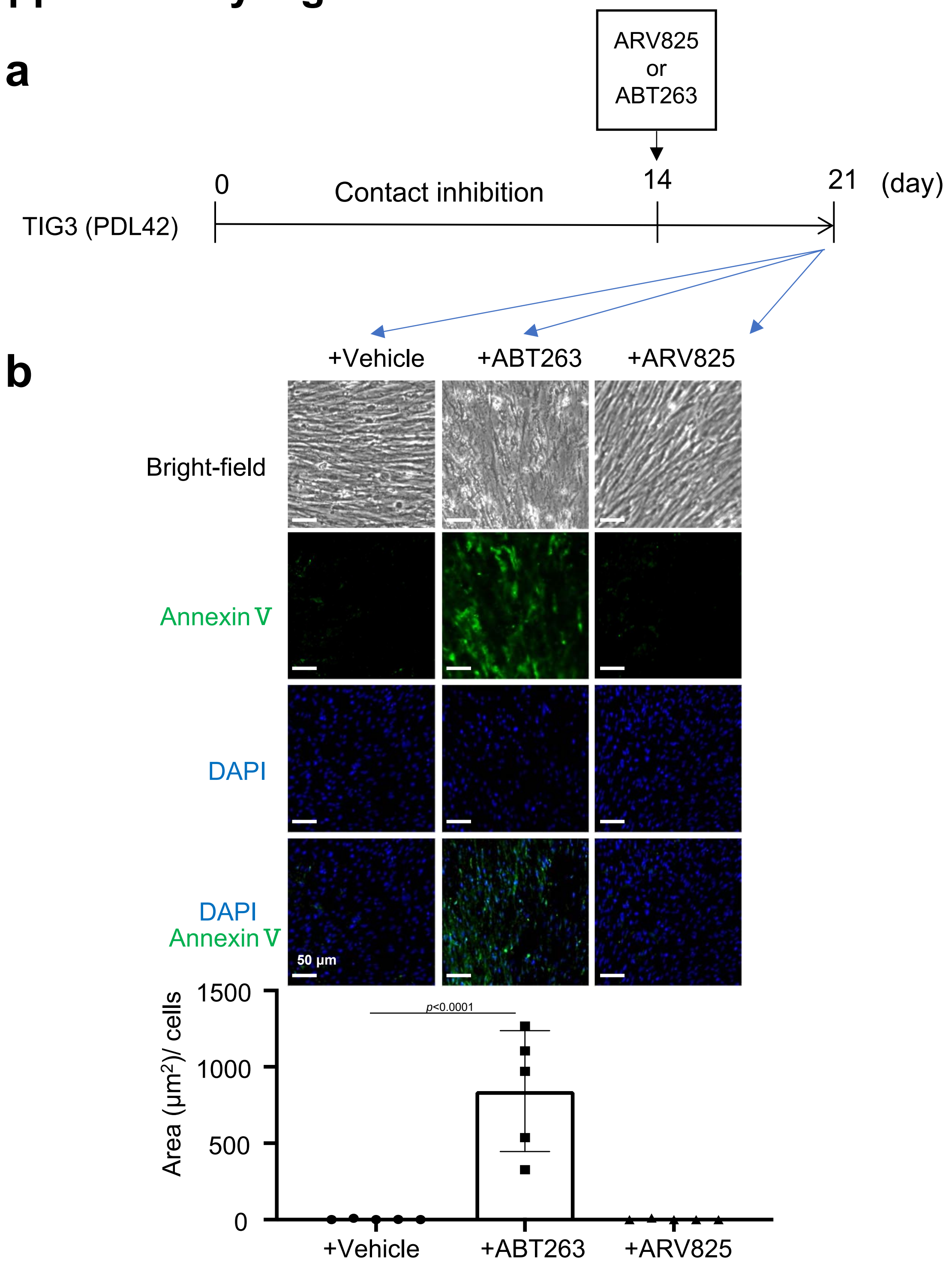

Supplementary Figure 3

ARV825 treatment does not induce apoptosis in quiescent cells. (**a**), Timeline of the experimental procedure. Pre-senescent TIG-3 cells were rendered quiescent by contact inhibition for 14 days, followed by treatment with 500 nM ABT263, 10 nM ARV825 or vehicle for 7 days. (**b**), These cells were subjected to AnnexinV staining assay for the detection of apoptotic cells. The number of AnnexinV positive area above threshold intensity was quantified and were shown at the bottom of the panel (**b**). Error bars indicate mean ± s.d. and the representative date from three independent experiments was shown (**b**). Statistical significance was determined with one-way ANOVA followed by Holm-Sidak multiple comparison test. *P* values <0.05 were considered significant.

Supplementary Fig.4

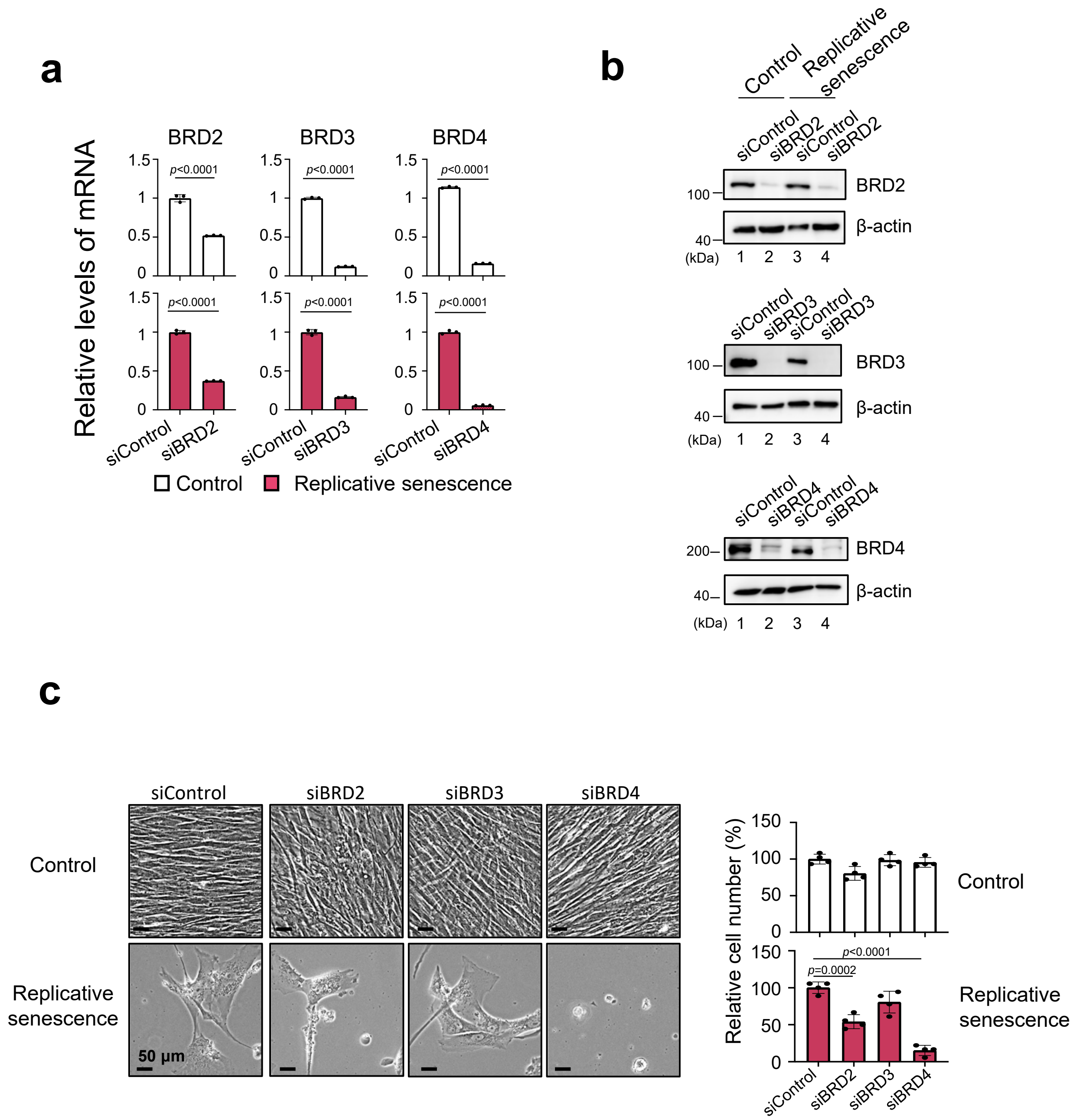

Supplementary Figure 4

ARV825-induced senolysis is phenocopied by BRD4 inhibition. Early passage (control) or late passage (replicative senescence) TIG-3 cells were transfected with previously validated siRNA oligos against BRD2, BRD3 and BRD4 or control siRNA oligo for twice at 2 days intervals. These cells were then subjected to RT-qPCR analysis (**a**), western blotting analysis using the antibodies shown on the right (**b**), or to the cell proliferation analysis (**c**). Representative photographs of the cells in the indicated culture conditions are shown and the histogram shown at the right of the panel indicates the relative cell number (**c**). For all graphs, error bars indicate mean ± s.d. and the representative date from three independent experiments was shown. Statistical significance was determined with two-tailed unpaired Student's t-test (**a**) or One-way ANOVA followed by Holm-Sidak multiple comparison test (**c**). *P* values <0.05 were considered significant.

Supplementary Fig.5

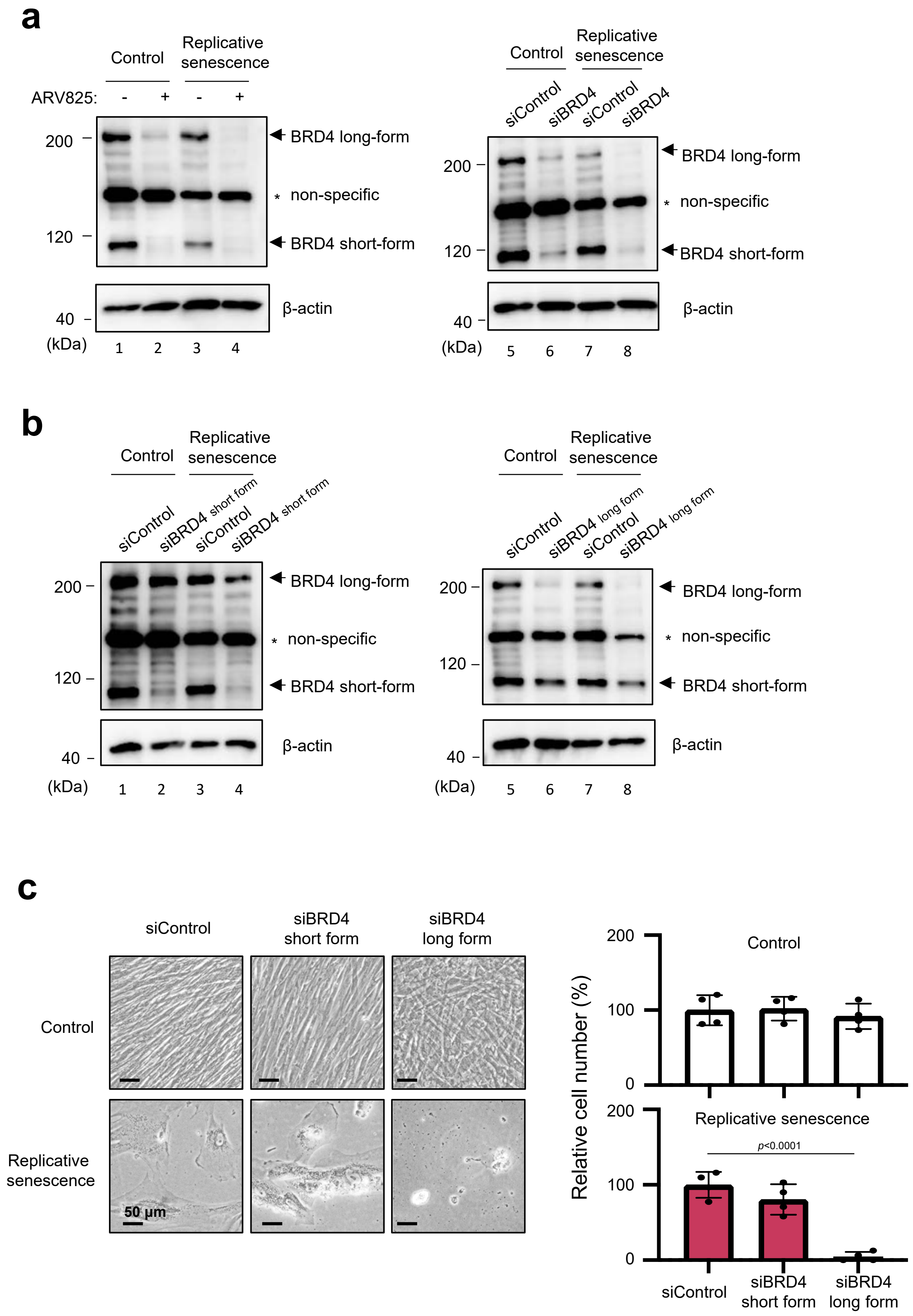

Supplementary Figure 5

BRD4 long isoform plays critical roles in preventing senescent cells from senolysis. Early passage (control) or late passage (replicative senescence) TIG-3 cells were treated with 10 nM ARV825 (left panel) or transfected with previously validated siRNA oligos against BRD4 or control siRNA oligo for twice at 2 days intervals (right panel). These cells were then subjected to western blotting analysis using the antibodies that detect all isoforms of BRD4 or β-actin (**a** and **b**), or to the cell proliferation analysis (**c**). Representative photographs of the cells in the indicated culture conditions are shown and the histogram shown at the right of the panel indicates the relative cell number (**c**). For all graphs, error bars indicate mean ± s.d.. The representative date from three independent experiments was shown and statistical significance was determined with one-way ANOVA followed by Tukey multiple comparison test (**c**). *P* values <0.05 were considered significant.

Supplementary Fig.6

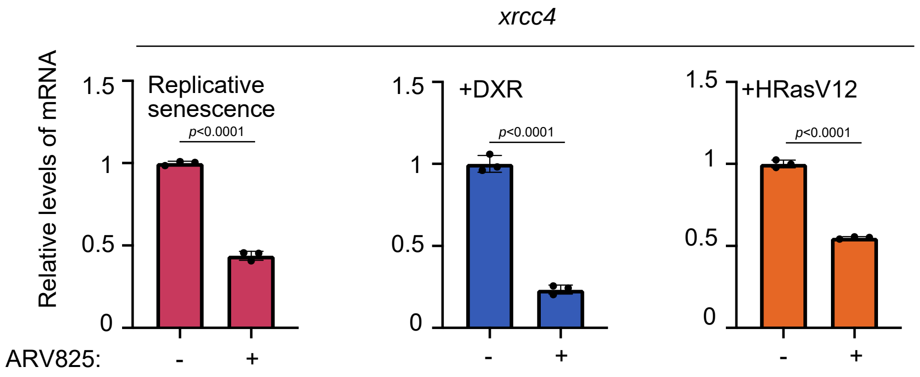

Supplementary Figure 6

ARV825 treatment reduces the expression of *xrcc4* gene in senescent cells. TIG3 cells were rendered senescence by serial passage (replicative senescence), DXR treatment (+DXR), or oncogenic Ras expression (+HRasV12) and were treated with 10 nM ARV825 or vehicle for 2 days. These cells were then subjected to RT-qPCR analysis for measuring the levels of *xrcc4* gene expression. The representative date from three independent experiments was shown and error bars indicate mean ± s.d.. Statistical significance was determined with two-tailed unpaired Student's t-test. *P* values <0.05 were considered significant.

Supplementary Fig.7

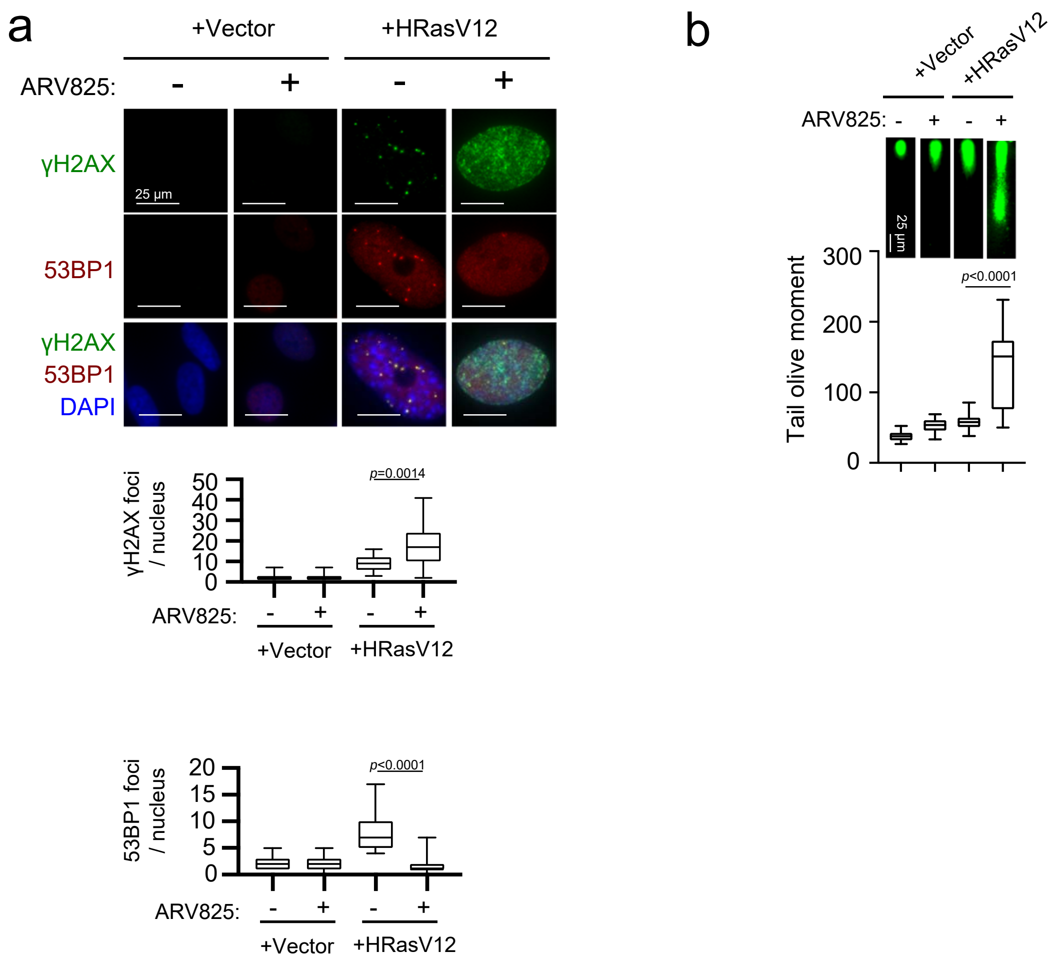

Supplementary Figure 7

ARV825 exacerbates DSBs in Ras-induced senescent cells. Early passage TIG-3 cells were rendered senescent by ectopic expression of oncogenic Ras (+HRasV12). These senescent cells and control pre-senescent cells (+Vector) were treated with 10 nM ARV825 (+) or vehicle (-) for 4 days and were then subjected to immunofluorescence staining using the antibodies shown on the left (**a**) or to neutral comet assay (**b**). The number of γH2AX or 53BP1 foci, above threshold intensity per nucleus (n=50) was quantified and was shown at the bottom of the panel (**a**). The average tail olive moments were shown at the bottom of the panel (**b**). Each box indicates the median and 25% and 75% quantiles, and the whiskers represent the minimum and maximum observations (**a and b**). The representative date from three independent experiments was shown and error bars indicate mean± s.d.. Statistical significance was determined with one-way ANOVA followed by Holm-Sidak multiple comparison test (**a** and **b**). *P* values <0.05 were considered significant.

Supplementary Fig.8

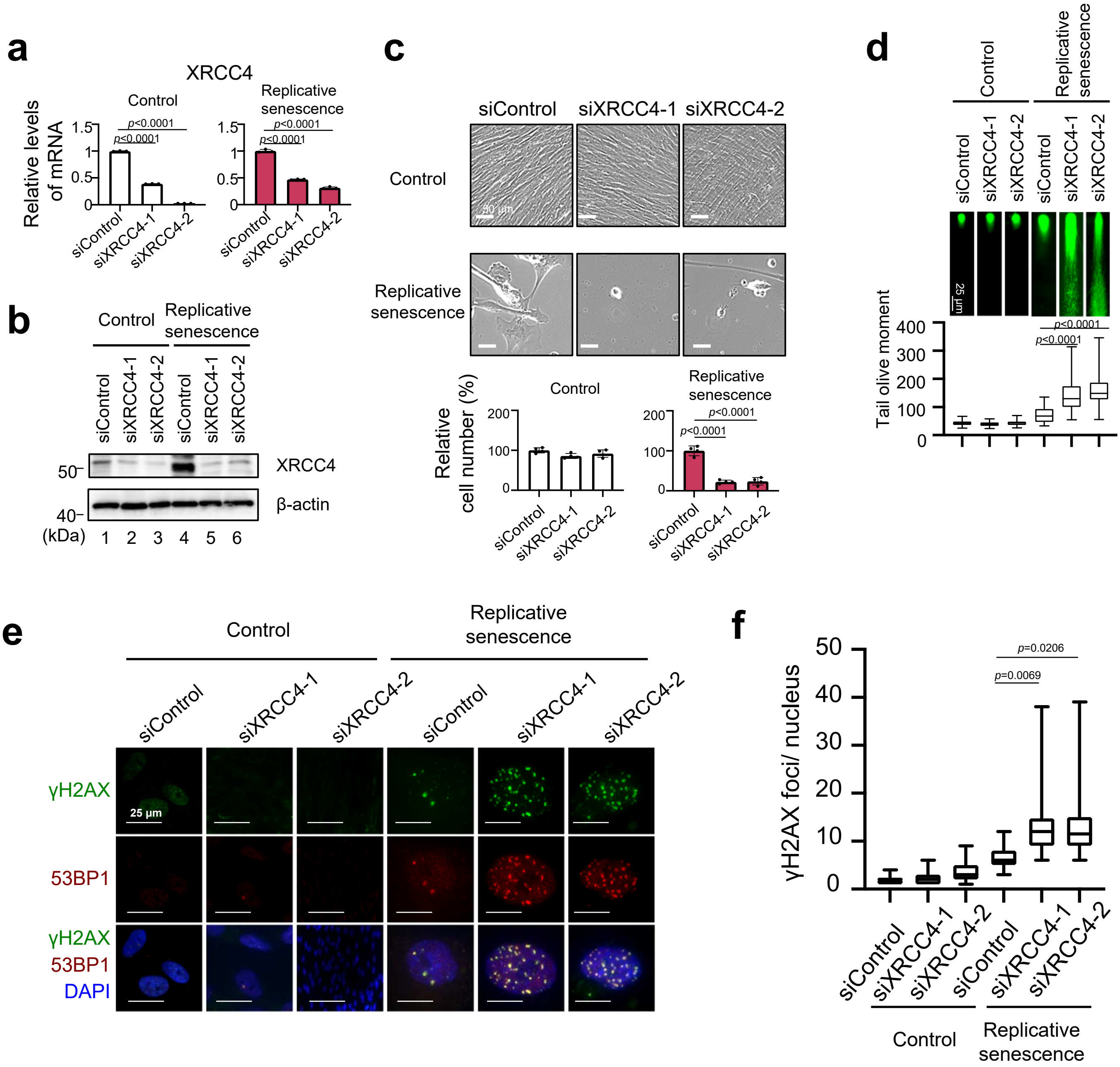

Supplementary Figure 8

siRNA-mediated depletion of XRCC4 exacerbated DSBs and cell death in senescent cells. Early passage (control) or late passage (replicative senescence) TIG-3 cells were transfected with previously validated two different siRNA oligos against *xrcc4* or control siRNA oligo for twice at 2 days intervals. These cells were then subjected to RT-qPCR analysis (**a**), western blotting analysis using the antibodies shown on the right (**b**), the cell proliferation analysis (**c**), neutral comet assay (**d**), or to the immunofluorescence staining using the antibodies shown on the left (**e and f**). Representative photographs of the cells in the indicated culture conditions are shown and the histogram shown at the bottom of the panel indicates the relative cell number (**c**). The average tail olive moments were shown at the bottom of the panel (**d**). The number of γH2AX foci above threshold intensity per nucleus (n=50) was quantified (**e and f**). Each box indicates the median and 25% and 75% quantiles, and the whiskers represent the minimum and maximum observations (**f**). For all graphs, the representative date from three independent experiments was shown. Error bars indicate mean± s.d.. Statistical significance was determined with two-tailed unpaired Student's t-test (**a**), one-way ANOVA followed by Tukey multiple comparison test (**c**), or one-way ANOVA followed by Holm-Sidak multiple comparison test (**d, f**). *P* values <0.05 were considered significant.

Supplementary Fig.9

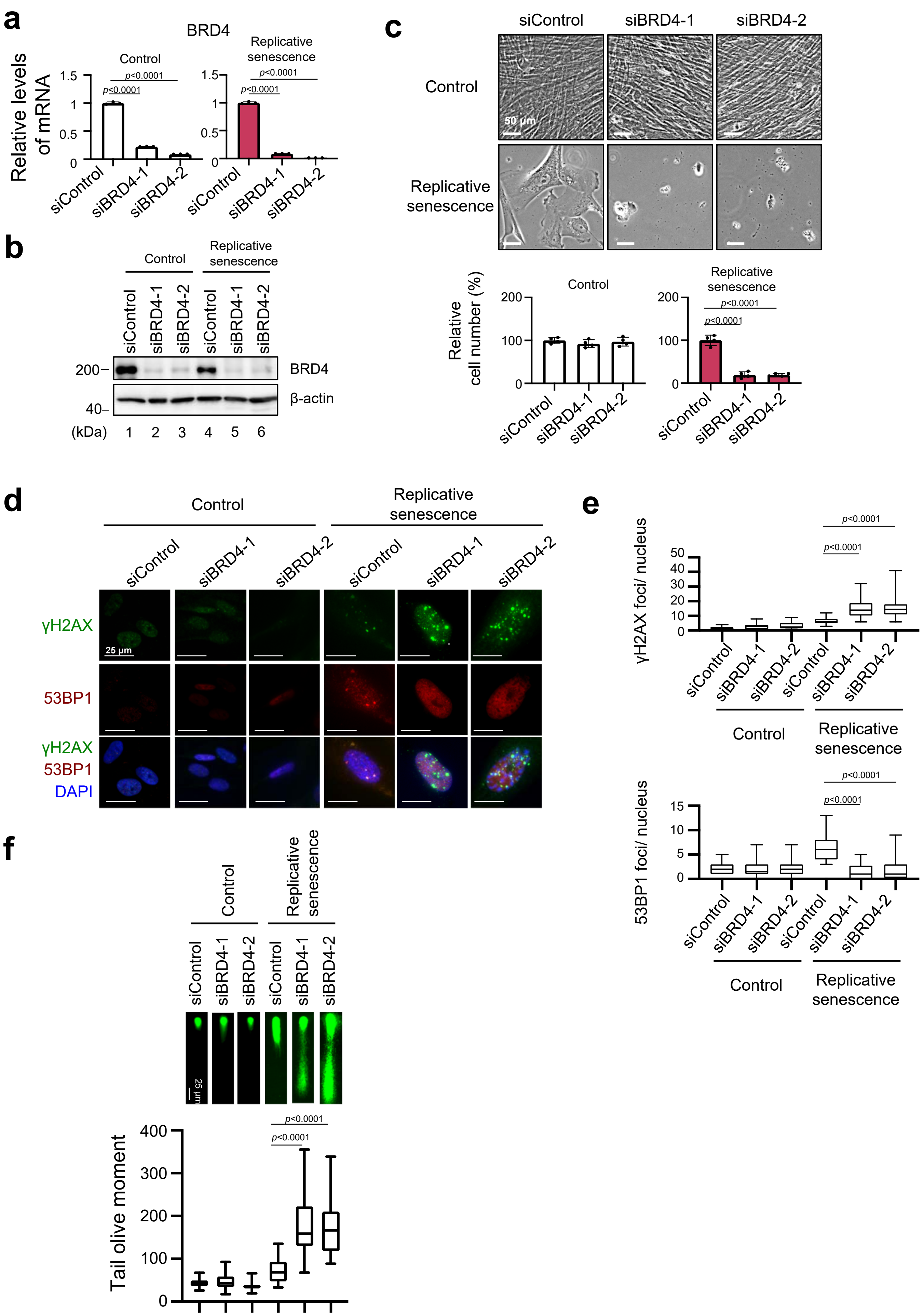

Supplementary Figure 9

The effects of ARV825 treatment were phenocopied by BRD4 inhibition in senescent cells. Early passage (control) or late passage (replicative senescence) TIG-3 cells were transfected with previously validated two different siRNA oligos against *brd4* or control siRNA oligo for twice at 2 days intervals. These cells were then subjected to RT-qPCR analysis (**a**), western blotting analysis using the antibodies shown on the right (**b**), the cell proliferation analysis (**c**), immunofluorescence staining using the antibodies shown on the left (**d and e**), or to neutral comet assay (**f**). Representative photographs of the cells in the indicated culture conditions are shown and the histogram shown at the bottom of the panel indicates the relative cell number (**c**). The number of γH2AX foci or 53BP1 foci, above threshold intensity per nucleus (n=50), was quantified (**e**). The average tail olive moments were shown at the bottom of the panel (**f**). Each box indicates the median and 25% and 75% quantiles, and the whiskers represent the minimum and maximum observations (**e and f**). For all graphs, error bars indicate mean ± s.d. and the representative date from three independent experiments was shown. Statistical significance was determined with two-tailed unpaired Student's t-test (**a**), one-way ANOVA followed by Tukey multiple comparison test (**c**), or one-way ANOVA followed by Holm-Sidak multiple comparison test (**e and f**). *P* values <0.05 were considered significant.

Supplementary Fig.10

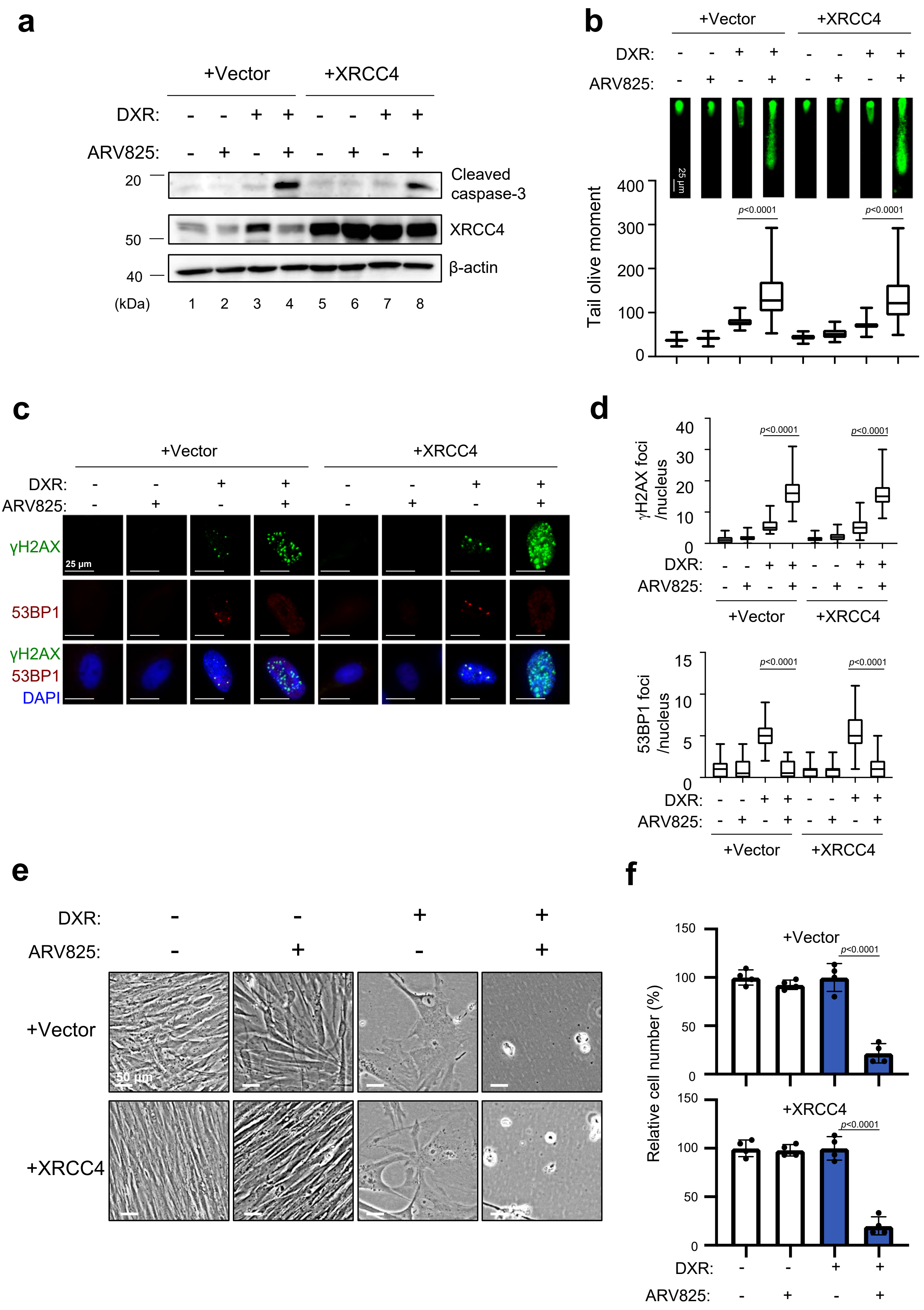

Supplementary Figure 10

Overexpression of XRCC4 failed to blunt senolytic effects of ARV825 in senescent cells. Early passage TIG3 cells were infected with retrovirus encoding XRCC4 or empty vector. These cells were treated with (+) or without (-) 250 ng/ml DXR for 10 days, followed by further treatment with 10 nM ARV825 (+) or vehicle (-) for 4 days. These cells were then subjected to western blotting (**a**), neutral comet assay (n=50) (**b**), the immunofluorescence staining using the antibodies shown on the left (**c and d**), or to the cell proliferation analysis (**e and f**). The average tail olive moments were shown at the bottom of the panel and each box indicates the median and the 25% and 75% quantiles, and the whiskers represent the minimum and maximum observations (**b**). The number of γH2AX foci or 53BP1 foci, above threshold intensity per nucleus (n=50), was quantified (**d**). Representative photographs of the cells in the indicated culture conditions are shown (**e**) and the histogram indicates the relative cell number (**f**). For all graphs, the representative date from three independent experiments was shown and error bars indicate mean ± s.d. Statistical significance was determined with one-way ANOVA followed by Holm-Sidak multiple comparison test (**b and d**), or one-way ANOVA followed by Tukey multiple comparison test (**f**). *P* values <0.05 were considered significant.

Supplementary Fig.11

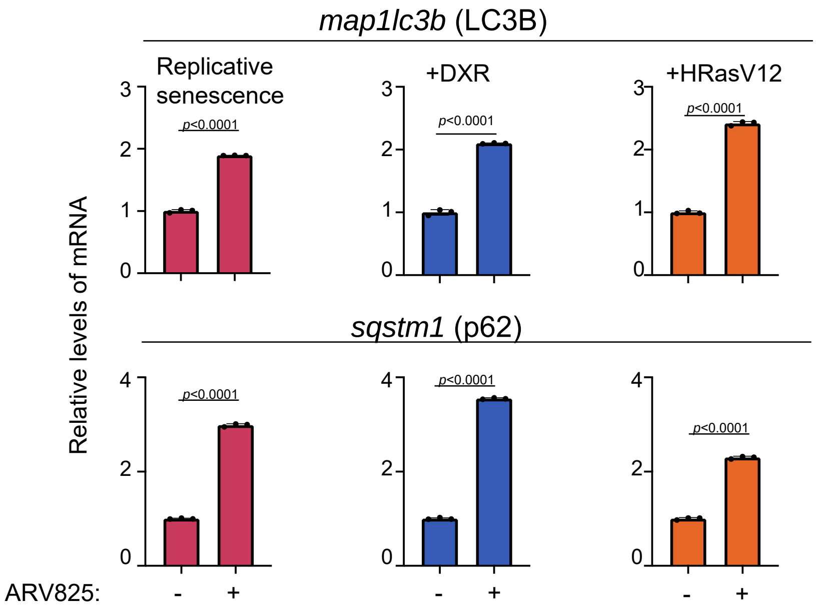

Supplementary Figure 11

ARV825 treatment upregulates the expression of autophagic genes in senescent cells. TIG3 cells were rendered senescence by serial passage (replicative senescence), DXR treatment for 10 days (+DXR), or oncogenic Ras expression (+HRasV12). These cells were then treated with 10 nM ARV825 or vehicle for 2 days and were subjected to RT-qPCR analysis for measuring the expression levels of *map1lc3b* (encoding LC3B) and *sqstm1* (encoding p62) genes. The representative date from three independent experiments was shown and error bars indicate mean ± s.d.. Statistical significance was determined with two-tailed unpaired Student's t-test. *P* values <0.05 were considered significant.

Supplementary Fig.12

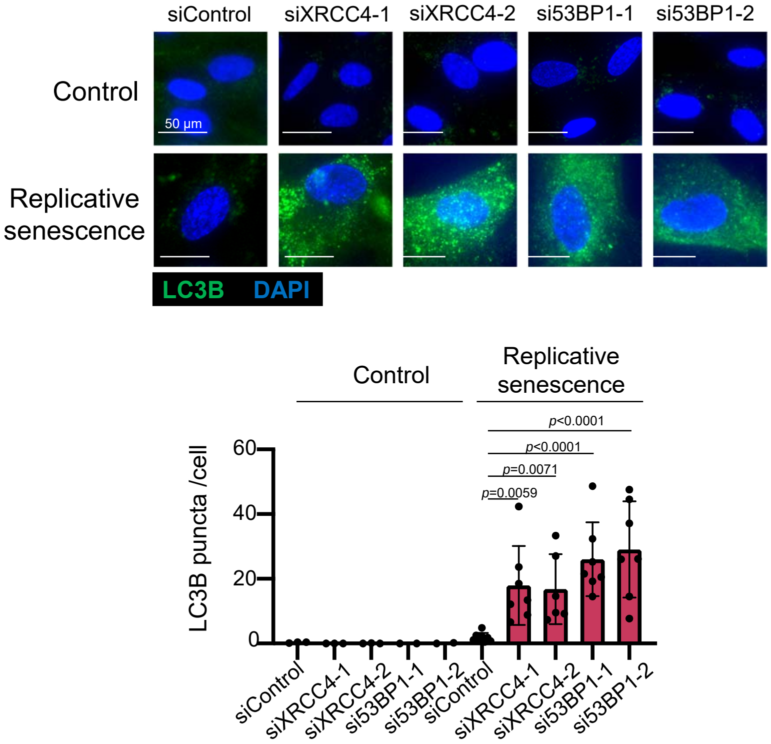

Supplementary Figure 12

siRNA-mediated depletion of XRCC4 or 53BP1 increases the signs of autophagy activation in senescent cells. Early passage (control) or late passage (replicative senescence) TIG3 cells were transfected with previously validated two different siRNA oligos against *xrcc4 or 53bp1* or control siRNA oligo for twice at 2 days intervals. These cells were then subjected to immunofluorescence staining using the antibody against LC3B (green). DNA was stained by DAPI (blue). The number of LC3B puncta above threshold intensity per cells (n=50) was quantified software and was shown at the bottom of the panel. Error bars indicate mean ± s.d. and the representative date from three independent experiments was shown. Statistical significance was determined with one-way ANOVA followed by Holm-Sidak multiple comparison test. *P* values <0.05 were considered significant.

Supplementary Fig.13

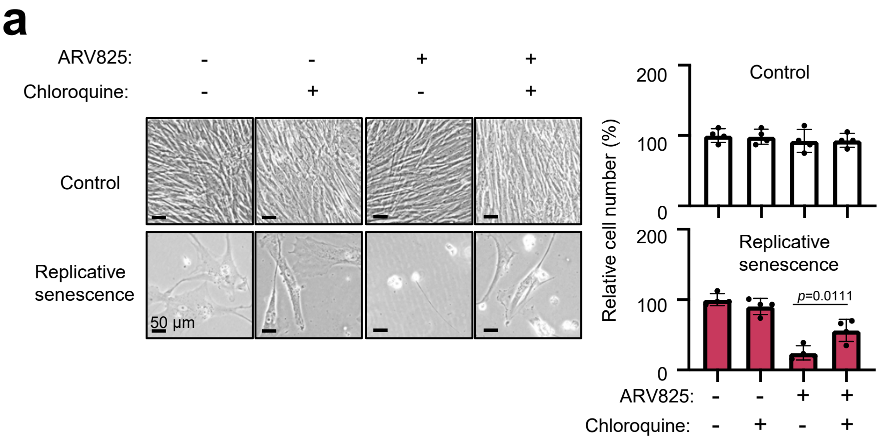

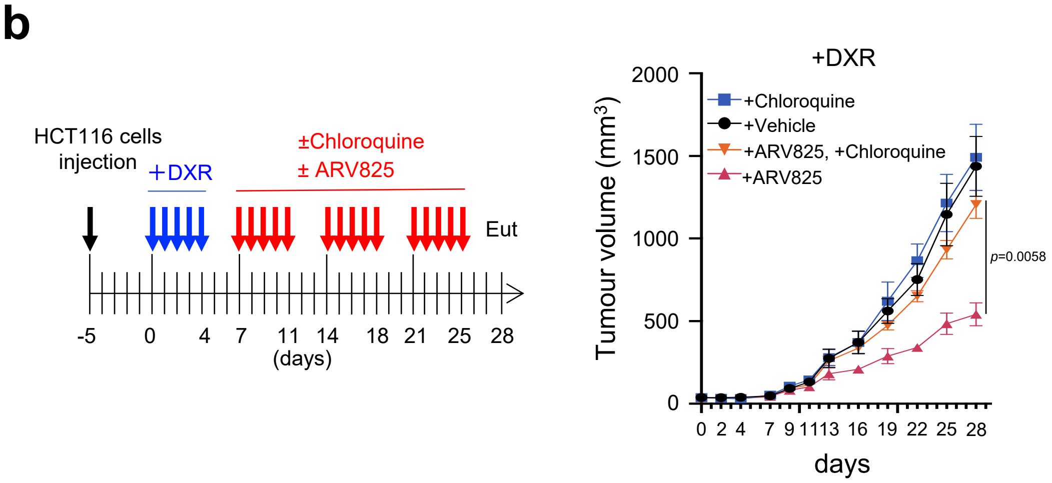

Supplementary Figure 13

Chloroquine attenuates the effects of ARV825-induced tumour growth suppression of xenografted tumours. Early passage (control) or late passage (replicative senescence) TIG-3 cells were treated with or without chemicals (10 nM ARV825, 500 nM chloroquine), as indicated at the top of the panel, for 4 days and were then subjected to the cell proliferation analysis (**a**). Representative photographs of the cells in the indicated culture conditions are shown and the histogram shown at the right of the panel indicates the relative cell number calculated from n=4 biologically independent cells (**a**). The representative date from three independent experiments was shown. And error bars indicate mean ± s.d. (**a**). (**b**), Timeline of the experimental procedure (n=5 per group). Blue arrow indicates the daily treatment with DXR (injected intraperitoneally with 1 mg/kg) for 5 consecutive days. Red arrow indicates a daily intraperitoneally injection with ARV825 (5 mg/kg) and/or Chloroquine (30 mg/kg) or vehicle for 5 consecutive days three times every 3 days. Tumour volume was calculated weekly from n=5 biologically independent animals per group using calipers (**b**). Error bars indicate mean ± s.e.m.(**b**). Statistical significance was determined with one-way ANOVA followed by Tukey multiple comparison test (**a**), or one-way ANOVA followed by Holm-Sidak multiple comparison test (**b**). *P* values <0.05 were considered significant.
