# Supplementary material for: A BET family protein degrader provokes senolysis by targeting NHEJ and autophagy in senescent cells
Source: Nat Commun. 2020 Apr 22;11:1935. doi: 10.1038/s41467-020-15719-6 (PMC7176673; doi:10.1038/s41467-020-15719-6)
Supplement: Supplementary file 3 — Reporting Summary [file 41467_2020_15719_MOESM3_ESM.pdf]

nature research

Corresponding author(s): Eiji Hara

Last updated by author(s): March 18, 2020

Reporting Summary

Nature Research wishes to improve the reproducibility of the work that we publish. This form provides structure for consistency and transparency in reporting. For further information on Nature Research policies, see Authors & Referees and the Editorial Policy Checklist .

Statistics

For all statistical analyses, confirm that the following items are present in the figure legend, table legend, main text, or Methods section.

n/a	Confirmed	
☐	☒	The exact sample size (*n*) for each experimental group/condition, given as a discrete number and unit of measurement
☐	☒	A statement on whether measurements were taken from distinct samples or whether the same sample was measured repeatedly
☐	☒	The statistical test(s) used AND whether they are one- or two-sided<br>*Only common tests should be described solely by name; describe more complex techniques in the Methods section.*
☒	☐	A description of all covariates tested
☒	☐	A description of any assumptions or corrections, such as tests of normality and adjustment for multiple comparisons
☐	☒	A full description of the statistical parameters including central tendency (e.g. means) or other basic estimates (e.g. regression coefficient) AND variation (e.g. standard deviation) or associated estimates of uncertainty (e.g. confidence intervals)
☐	☒	For null hypothesis testing, the test statistic (e.g. *F*, *t*, *r*) with confidence intervals, effect sizes, degrees of freedom and *P* value noted<br>*Give P values as exact values whenever suitable.*
☒	☐	For Bayesian analysis, information on the choice of priors and Markov chain Monte Carlo settings
☒	☐	For hierarchical and complex designs, identification of the appropriate level for tests and full reporting of outcomes
☒	☐	Estimates of effect sizes (e.g. Cohen's *d*, Pearson's *r*), indicating how they were calculated

Our web collection on statistics for biologists contains articles on many of the points above.

Software and code

Policy information about availability of computer code

Data collection	Microscopic data: All-in-One Fluorescence Microscope (BZ-710; keyence)<br>Immunoblotting data: LAS-3000mini imaging system (Fujifilm)<br>Quantitative real-time PCR data: StepOnePlus PCR system (Applied Biosystems)<br>High throughput screening data: Envision plate reader (Perkinelmer)<br>RNA sequencing data: Illumina HiSeq 3000 platform (Illumina)
Data analysis	Statistical analysis: GraphPad Prism 8, Excel2016<br>Immunofluorescence staining and immunohistochemistry staining analysis: BZ-X700 analyzer software<br>Immunoblotting analysis: Multi Guage V 3.1 (Fujifilm)<br>Comet assay analysis: CometScore2.0<br>RNA sequencing analysis: Illumina Casava ver.1.8.2 software (Illumina), TopHat ver. 2.0.13, Bowtie2 ver. 2.2.3, SAMtools ver. 0.1.19, Cufflinks ver. 2.2.1

For manuscripts utilizing custom algorithms or software that are central to the research but not yet described in published literature, software must be made available to editors/reviewers. We strongly encourage code deposition in a community repository (e.g. GitHub). See the Nature Research guidelines for submitting code & software for further information.

Data

Policy information about availability of data

All manuscripts must include a data availability statement. This statement should provide the following information, where applicable:

- Accession codes, unique identifiers, or web links for publicly available datasets
- A list of figures that have associated raw data
- A description of any restrictions on data availability

RNA sequencing data of Fig.3a has been deposited in the Gene Expression Omnibus under accession number GSE140961. The source data underlying Figs 1b-e, 2b-

e, 2g and h, 3b-I, 4a-f and Supplementary Figs. 1, 2, 3b, 4, 5, 6, 7, 8, 9, 10, 11, 12 and 13 are provided as a Source Data file.

Field-specific reporting

Please select the one below that is the best fit for your research. If you are not sure, read the appropriate sections before making your selection.

- [x] Life sciences
- [ ] Behavioural & social sciences
- [ ] Ecological, evolutionary & environmental sciences

For a reference copy of the document with all sections, see nature.com/documents/nr-reporting-summary-flat.pdf

Life sciences study design

All studies must disclose on these points even when the disclosure is negative.

Sample size

The sample size used in this study was determined based on the expense of data collection, and the need to have sufficient statistical power.

Data exclusions

No data was excluded from the analysis.

Replication

All data presented were obtained from three or two independent experiments with similar outcomes. (see Figure legends and Methods)

Randomization

For all vivo experiments, animals were randomly assigned to experimental groups. For in vitro experiments, wells were randomly assigned as control or treated samples.

Blinding

Data collection and Analysis were not performed blind. Controls and relative group samples were processed either simultaneously or in parallel in all experiments

Reporting for specific materials, systems and methods

We require information from authors about some types of materials, experimental systems and methods used in many studies. Here, indicate whether each material, system or method listed is relevant to your study. If you are not sure if a list item applies to your research, read the appropriate section before selecting a response.

Materials & experimental systems

n/a	Involved in the study	
☐	☒	Antibodies
☐	☒	Eukaryotic cell lines
☒	☐	Palaeontology
☐	☒	Animals and other organisms
☒	☐	Human research participants
☒	☐	Clinical data

Methods

n/a	Involved in the study	
☒	☐	ChIP-seq
☒	☐	Flow cytometry
☒	☐	MRI-based neuroimaging

Antibodies

Antibodies used

α-SMA (Sigma, cat#: A5228), β-Actin (Sigma, cat#: A5316), BRD2 (Abcam, cat#: ab139690), BRD3 (Santa cruz, cat#: sc-81202), BRD4 (Cell signaling, cat#: 13440 or abcam, cat#: ab128874), Cleaved caspase3 (Cell signaling, cat#: 9664), Gro-α (Abcam, cat#: ab17882), γH2AX (Millpore, cat#: 05-636 or abcam, cat#:ab2893), IL-6 (Abcam, cat#: ab6672), LaminB1 (Abcam, cat#: ab16048), LC3B (Cell signaling, cat#: 2775), p21 (Cell signaling, cat#: 2947 or Abcam, cat#: ab107099 or Abcam, cat#: ab2961), XRCC4 (Santa cruz, cat#: sc-271087), 53BP1, (Novus biologicals, cat#: NB100-304 or Santa cruz, cat#: sc-22760)

Validation

All antibodies used in this study were commercially available antibodies and were validated by companies. Data sheet is available from the web links as described below.

α-SMA; https://www.sigmaaldrich.com/catalog/product/sigma/a5228?lang=ja&region=JP

β-Actin; https://www.sigmaaldrich.com/catalog/product/sigma/a5316?lang=ja&region=JP

BRD2; https://www.abcam.com/brd2-antibody-epr7642-ab139690.html

BRD3; https://www.scbt.com/scbt/product/brd3-antibody-2088c3a

BRD4; https://www.cellsignal.com/products/primary-antibodies/brd4-e2a7x-rabbit-mab/13440

BRD4; https://www.abcam.co.jp/brd4-antibody-epr51502-ab128874.html

Cleaved caspase3; https://www.cellsignal.jp/products/primary-antibodies/cleaved-caspase-3-asp175-5a1e-rabbit-mab/9664

Gro-α; https://www.abcam.co.jp/gro-alpha-antibody-ab17882.html

γH2AX; http://www.merckmillipore.com/JP/ja/product/Anti-phospho-Histone-H2A.X-Ser139-Antibody-clone-JBW301,MM_NF-05-636-I?ReferrerURL=https%3A%2F%2Fwww.google.com%2F&bd=1

γH2AX; https://www.abcam.co.jp/gamma-h2ax-phospho-s139-antibody-ab2893.html

IL-6; https://www.abcam.co.jp/il-6-antibody-ab6672.html

LaminB1; https://www.abcam.co.jp/lamin-b1-antibody-nuclear-envelope-marker-ab16048.html

LC3B; https://www.cellsignal.jp/products/primary-antibodies/lc3b-antibody/2775
p21; https://www.cellsignal.jp/products/primary-antibodies/p21-waf1-cip1-12d1-rabbit-mab/2947
p21; https://www.abcam.co.jp/p21-antibody-hugo291-ab107099.html
p21; https://www.abcam.co.jp/p21-antibody-ab2961.html
XRCC4; https://www.scbt.com/scbt/ja/product/xrcc4-antibody-c-4?requestFrom=search
53BP1; https://www.novusbio.com/products/53bp1-antibody_nb100-304
53BP1; https://www.scbt.com/scbt/product/53bp1-antibody-h-300

Eukaryotic cell lines

Policy information about cell lines

Cell line source(s)	TIG-3, hRPE and HCT116 cells were obtained from Japanese Cancer Research Resources Bank (JCRB), Lonza Inc., and ATCC, respectively. Mouse embryonic fibroblasts (MEFs) were established from day 13.5 mouse embryos. Mouse primary hepatic stellate cells (mHSCs) were isolated from mouse liver as described in the Method section. IMR-90 ER:RAS cells were established as described in Barradas et al., Genes Dev., 23, 1177-1182 and were provided by Dr. Gordon Peters.
Authentication	Since MEF and mHSC were developed in our lab, authentication was not necessary. Other cells were obtained from public bioresources bank or Company and were not authenticated by ourselves.
Mycoplasma contamination	We have confirmed that there were not mycoplasma contamination in our tissue culture cells and were stated in "Cell culture section" of the METHOD page.
Commonly misidentified lines (See ICLAC register)	No commonly misidentified cell lines were used in this study

Animals and other organisms

Policy information about studies involving animals; ARRIVE guidelines recommended for reporting animal research

Laboratory animals	10-16 weeks old female pregnant C57/BL6 mice and 4-5 weeks old female Nude (nu/nu) mice were purchased from Charles River Laboratories Japan, Inc.
Wild animals	The study did not involve wild animals.
Field-collected samples	The study did not involve samples collected from the field.
Ethics oversight	All mouse experiments were approved by the Animal Research Committee of Research Institute for Microbial Diseases, Osaka University.

Note that full information on the approval of the study protocol must also be provided in the manuscript.
